# Progress in the Synthesis of Colloidal Machines

**DOI:** 10.1021/accountsmr.3c00203

**Published:** 2024-02-22

**Authors:** Nicolle S. Jackson, Samira Munkaila, Lasya Damaraju, Marcus Weck

**Affiliations:** Molecular Design Institute and Department of Chemistry, New York University, New York, New York 10003, United States

## Abstract

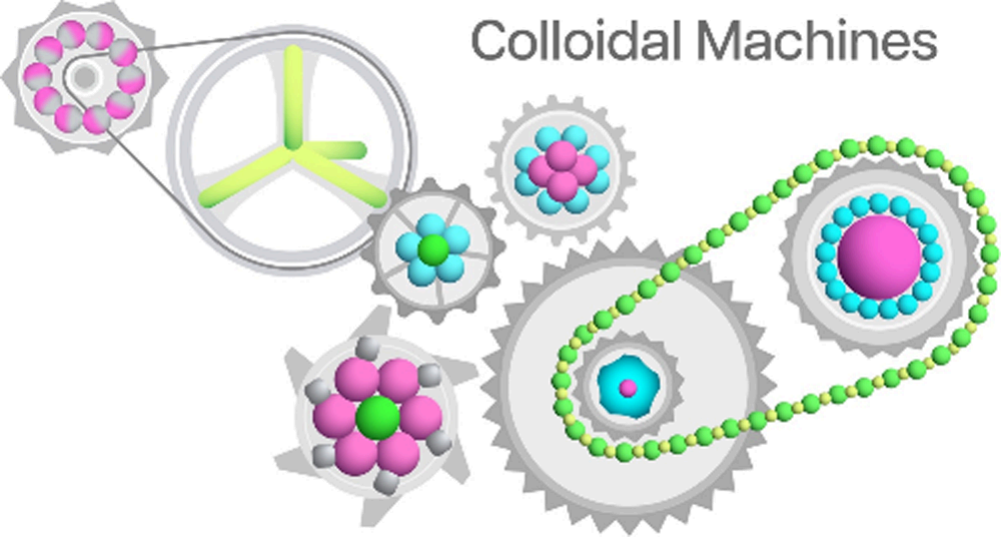

For the past decade, the field of colloidal science has expanded the collection of colloidal particles to include an entire library of subunits that can be isotropic or anisotropic in terms of structural morphology or chemical composition. Using anisotropic subunits, the field has assembled a variety of static and dynamic structures. For this Account, we use the umbrella term “dynamic colloids” to describe subunits capable of movement, shape-shifting, or any other type of action in response to a stimulus and “static colloids” to describe those that are unresponsive to such stimuli. We view dynamic colloids as an access point to colloidal machines, a unique and emerging subfield of machines, and colloidal science. The assembly of dynamic subunits into colloidal machines differs from traditional self-assembly only in the final structures assembled, not the methods used. Dynamic assemblies have the capacity to interact with their environment in ways that traditional anisotropic self-assemblies do not. Here, we present the current state of the field of colloidal science toward the introduction of the next wave of colloidal machines.

Machines are ubiquitous in nature and synthetic systems, governing every aspect of life. In mechanics, a machine is a device that transmits or modifies force or motion. In biology, nature’s machines such as kinesin or ATP synthetase are essential to life. In the synthetic realm, molecular machines and nanomachines, recognized with the Nobel prize, include diverse systems, such as molecular rotors and elevators fabricated using bottom-up synthetic methods. On the microscale, microscopic motors based on microelectromechanical systems (MEMs) have been achieved via top-down methods such as micromachining. On the colloidal scale, machines are conspicuously absent due, in part, to the difficulty in navigating combinatory design spaces. We view colloidal machines (100 nm to 10 μm) as the next line of miniaturization in machines. Due to the bottom-up fabrication methods generally used in creating dynamic colloids, one can achieve complexity at a smaller scale than possible with top-down approaches. The introduction of colloidal scale machines would bridge the gap between the microscopic world with its macroscopic counterparts, the nanoworld with its molecular machines, and the biological world with nature’s machinery.

Reported colloidal machines to date are apparatuses that consist of multiple components of a single composition of dynamic subunits that come together to perform some work. The next step toward complex colloidal machines is systems containing *multiple dynamic colloidal scale components* that come together to act in tandem to perform some work on the surrounding environment. We envision repurposing a library of dynamic particles originally intended to be used as anisotropic subunits into dynamic components of a colloidal machine. Computationally, the idea of colloidal machines has been extensively explored; however, synthetically, there has been limited exploration. In order to implement this existing library into colloidal machines, the key next step is the development of *synthetic combinatorial design spaces*.

## Introduction

As technology advances and miniaturizes, bottom-up techniques to build micro- or nanoscale components are increasing. Bottom-up approaches to materials synthesis allow for a high degree of control over the structure and composition of the final material and its properties. In the colloidal domain, bottom-up fabrication strategies use subunits consisting of polymer, metallic, or inorganic particles with sizes between 100 nm and 10 μm to build hierarchical superstructures. Additionally, because subunits can be fabricated with intrinsic properties such as magnetism or dielectricity, they can be directed to assemble, potentially, *in situ*.

To access complex structures on the colloidal scale through bottom-up design, different subunits are required that endow colloids with tunable sizes, shapes, and chemical compositions. Extending the ability to fine-tune the structural or chemical composition during the synthesis is critical to building more complex superstructures. This strategy allows dynamic assemblies to be achieved through a rational particle design rather than complex functional group synthesis and surface modification. Previous reports of assemblies focused on static subunits due to their facile synthetic methods.^1^ Static subunits, however, lack a responsiveness to external stimuli. We view dynamic colloids as the logical subunits to overcome this challenge.

In addition to dynamic subunits, achieving complex responsive/dynamic assemblies necessitates chemical or structural anisotropy. Isotropic spherical colloids usually only adopt closed-packed assemblies.^2^ Thus, introducing anisotropy opens the path to achieving complex and open-packed hierarchical assemblies. Table 1 highlights some of the anisotropic colloidal material compositions, shapes, and synthetic methods reported to date. Introducing responsiveness to external stimuli allows dynamic colloids to impart functionality to colloidal assemblies, opening an avenue to create machine-like colloidal structures.^3^

**Table 1:**
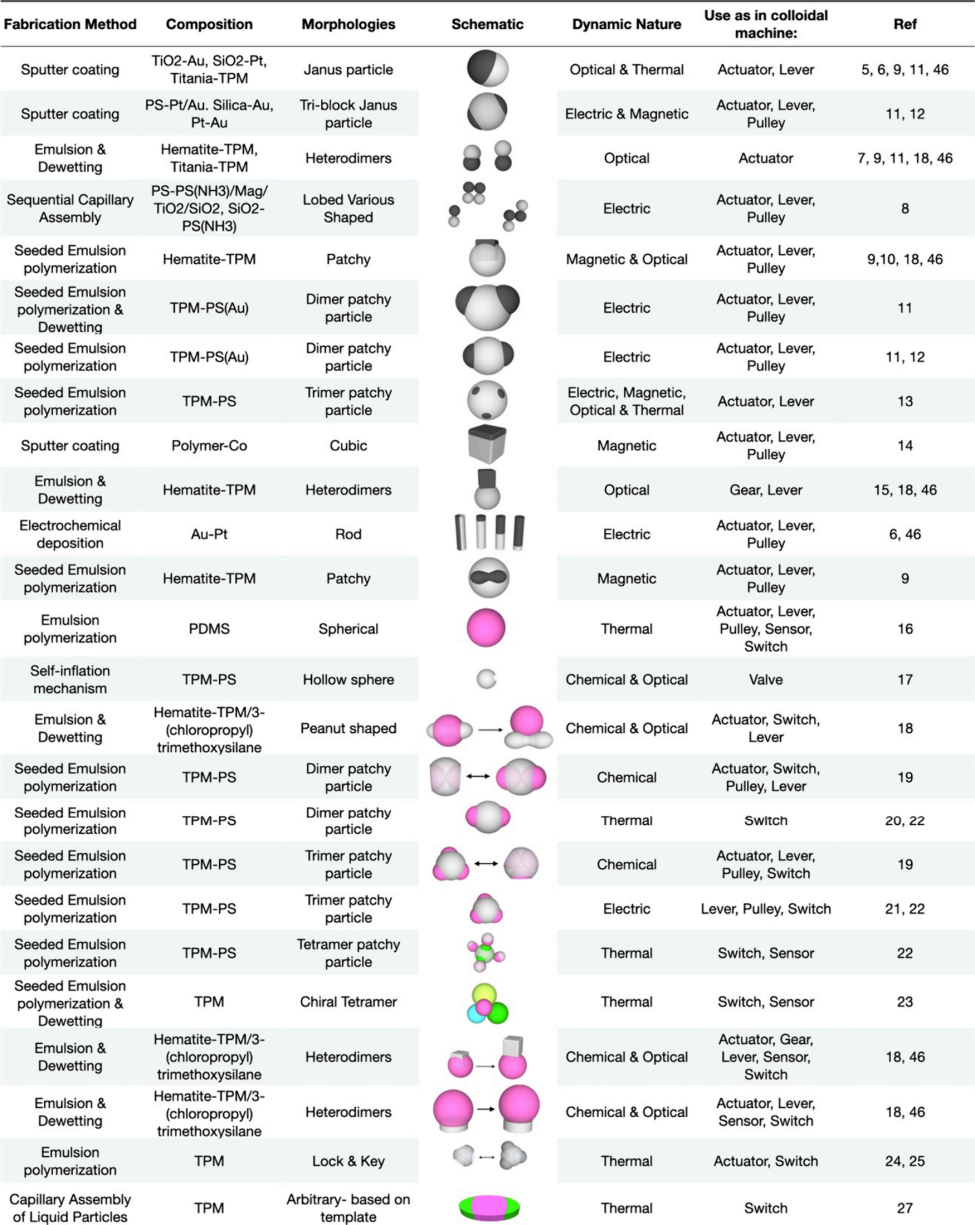
Library of Subunits^a^

a Detailing the fabrication method used in synthesis, the composition, the resultant morphology, schematic illustration of the particle, dynamic nature, and potential use as a machine. The potential uses listed in the table are Actuator: a device that converts energy into motion such as push, lift, pull, rotate, or open/close. Sensor: detect and measure physical properties or conditions. Lever: pivots around a fixed point, amplifies an input force to provide a greater output force. Gear: transmit motion (torque and speed) between different parts of a machine (type of lever). Pulley: changes direction of a force or object. Switch: can disconnect or connect a path. Valve: controls movement/flow through a confined space.

A colloidal machine based on dynamic subunits can transmit force or direct the application of a force by responding to stimuli (input) to perform a work (output). While the fabrication of colloidal machines is only in its infancy, they have already demonstrated promising potential in applications such as soft robotics, sensors, biomedical devices, and electronic components. Given the current progress toward colloidal machines, this Account aims to provide an overview of synthetically feasible dynamic colloids. We discuss the properties of dynamic colloids and emphasize overarching concepts, trends, and potential areas of future research. Various assembly strategies of dynamic colloids into dynamic structures are presented with a discussion of their assembly mechanisms. Potential applications are highlighted with an emphasis on practical barriers to using colloidal machines. We present here the use of published subunits not as originally intended but reimaged for use in colloidal machines.

## Guiding Design Principles

Designing and creating materials with properties that enable multifunctionality often requires structural control on multiple scales. To optimize this design process, a bottom-up synthetic strategy that focuses on the design and synthesis of subunits followed by their assembly into larger structures is ideal.^4^ The key benefit to employing a bottom-up approach is that it offers precise control over the structure’s final properties. Using one or more of the subunits described in Table 1 allows for assembly of an assortment of structures with various chemical and physical properties as well as various responsive natures.^5−27^

A bottom-up approach can be used to design dynamic colloidal particles, but a thorough exploration of the composition of these particles and their dynamic nature is required for their implementation into colloidal machines. All colloidal particles exhibit Brownian motion due to their small size (100 nm to 10 μm), resulting from the *random* momentum transfer to the particles from the thermal motion of the molecules of the suspending medium. Given that this force is random, stable assemblies form only when the driving force for assembly *overcomes* Brownian motion and any other repulsive forces. The use of physicochemical interactions is an important part of all colloidal assemblies. However, they are not the focus of this Account. An in-depth discussion of these interactions and their resulting assemblies can be found in the literature.^28,29^ Instead, we focus on how external stimuli can be used as functional handles to assemble dynamic subunits.^29,30^

External forces such as magnetic, electric, thermal, optical, pressure, acoustic, and radiative fields can be used to assemble colloidal superstructures.^29−31^ Field strength, geometry, and frequency can be modified to tune colloidal interactions.^30^ External fields affect colloidal suspensions according to the intrinsic properties of the particles and the medium. For example, magnetic and electric fields typically use dipole–dipole interactions inherent to or induced by the field to direct subunit assembly. Thus, the subunits will align to form superstructures while the fields are active and degrade back into colloidal suspensions when the field is turned off.^32^

A thermal external stimulus is employed to control assemblies based on DNA hybridization. Particles can be coated or covalently functionalized with DNA and assemblies finely controlled based on solution temperature.^2,16,20,22,29^ Another method to control these systems is DNA strand displacement,^33^ allowing for the introduction of additional phase transitions and reconfigurations to be incorporated into the system. Temperature modulation influences assembly based on the discrete melting points of the incorporated DNA strands.

Optical and chemical stimuli can be used at the fluid interfaces, termed the triple phase boundary, to alter the interfacial energies of components. The origin of the interfacial energies of components within the system is derived from minimizing the total surface energy between the different phases.^28^ Thus, the triple-phase boundary can be adjusted by altering the interfacial energies of the phases. This is achieved chemically by altering the affinity between components of the subunits, generally causing a shape change, which, in turn, represents the physical modification of the triple-phase boundary. Contrastingly, the triple-phase boundary can be optically altered by a chemical reaction triggered by an optical stimulus of a photoactivated component (like the modified photo-Fenton reaction) in the subunit.^34^

For the bottom-up design of materials, the general principles discussed here should be considered in addition to the inherent physiochemical interactions in the design fabrication of subunits. These principles, such as external stimuli, should guide the subunit composition when designing the system. External stimuli forces can be either universal (ex: electric, magnetic) or directed (ex: optical) but require specific compositions of subunits to access these types of controls. All of these techniques can create useful functional handles in assemblies with the proper design of the individual subunits and systems.

## Dynamic Colloids

Dynamic colloids are key subunits for colloidal machines due to their ability to access structures and materials with unique properties, including a wide range of responses to optical, electric, magnetic, thermal, or chemical external stimuli. We differentiate dynamic colloids from static colloids through their interactions with an applied stimulus. For example, dipatch particles that have nonfunctionalized and functionalized patches behave differently in an applied electric field on account of their varied interactions. Nonfunctionalized patches interact with the field based on the symmetry of the particle; functionalized patches, on the other hand, interact with the field based on patch-field interactions and the symmetry of functionalized patches. The unique behavior of dynamic colloids offers several advantages over primitive colloids, including responsiveness, reversibility, and tunability. Certain limitations, however, including stability, complex synthesis, and scalability can hinder their use for specific applications.^25^

**Figure 1:**
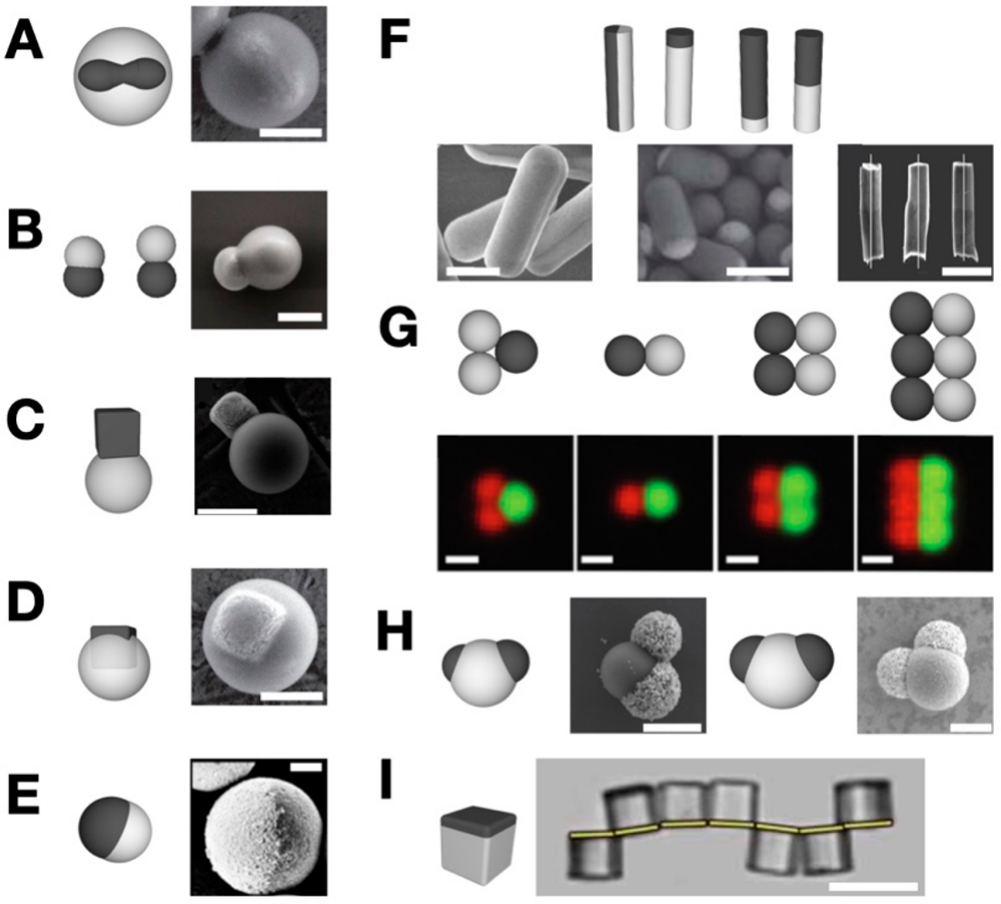
Active subunits. The subunits are represented by their schematics and corresponding scanning electron microscope (SEM) images: (A) Peanut-shaped hematite particle embedded in a TPM sphere. Reproduced with permission from ref (9). Copyright 2014 The Authors. (B–D) heterodimers of TPM and hematite. Reproduced with permission from refs (7), (9), and (15). Copyright 2021, 2014, and 2018 The Authors. (E) Spherical gold and titania Janus particle. Reproduced with permission from refs (5) and (6). Copyright 2020 The Authors and Copyright 2017 Elsevier. (F) Active colloidal rods with gold and platinum segments. Reproduced with permission from refs (25), (37), and (39). Copyright 2013 Royal Society of Chemistry, Copyright 2013 American Physical Society, and Copyright 2013 Springer Nature. (G) Colloidal poly(styrene) microspheres embedded with different magnetic nanoparticles. Reproduced with permission from ref (8). Copyright 2017 Royal Society of Chemistry. (H) Di-patchy particles of different symmetries selectively coated with gold. Reproduced with permission from refs (11) and (12). Copyright 2019 American Chemical Society and Copyright 2021 American Chemical Society. (I) Cube-shaped patchy particle with one side coated with cobalt. Reproduced with permission from ref (14). Copyright 2017 The Authors. (A)–(H) Scale bars 1 μm; scale bar for (I) 20 μm.

**Figure 2:**
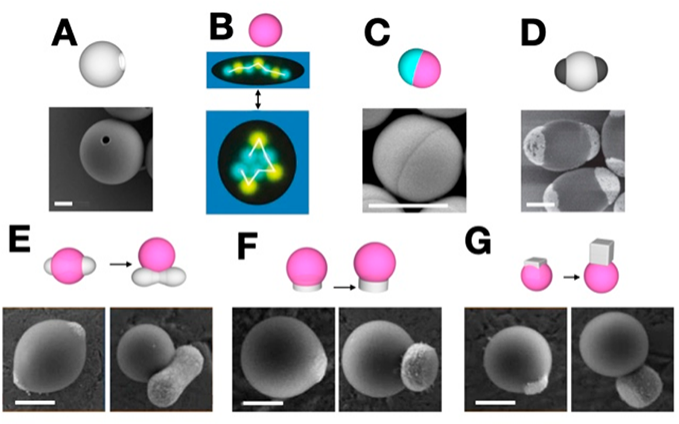
Stimulus responsive subunits. These subunits respond to external stimuli, such as temperature, pH, light, electric, or magnetic fields. The subunits are represented by their schematics and corresponding SEM images: (A) TPM colloidal microcapsules. Reproduced with permission from ref (17). Copyright 2021 Springer Nature. (B) Colloidal droplets selectively labeled with DNA strands forming an alternating colloidal droplet chain, image falsely colored for clarity. Reproduced with permission from ref (16). Copyright 2022 The Authors. (C) Janus particle with different DNA-coated patches. Reproduced with permission from ref (40). Copyright 2020 American Chemical Society. (D) Metallodielectric di-patch particles with selectively gold coated patches. Reproduced with permission from ref (12). Copyright 2021 American Chemical Society. (E) Shape-shifting particles of peanut-shaped hematite and TPM. Reproduced with permission from ref (18). Copyright 2016 The Authors. (F,G) Shape-shifting particles of disc and cubic hematite and TPM;. Reproduced with permission from ref (18). Copyright 2016 The Authors. All scale bars 1 μm.

**Figure 3:**
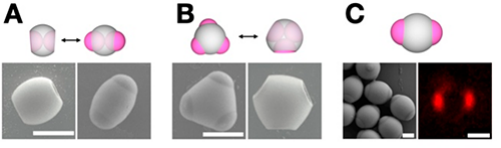
Dynamic colloids from the Weck group. The subunits are represented by their schematics and corresponding SEM or confocal images: (A) shape-shifting dipatch particle; (B) tripatch particle shifting between concave and convex features. (A, B) Reproduced with permission from ref (19). Copyright 2017 Wiley-VCH Verlag GmbH & Co. KGaA. (C) Di-patchy particles composed of PS-TPM-PS. Reproduced with permission from ref (20). Copyright 2021 John Wiley and Sons. All scale bars 1 μm.

We subdivide dynamic colloids based on the nature of their response into active colloids (Figure 1) and stimulus-responsive colloids (Figure 2). We term “active colloids” as those that respond to external stimuli by generating motion such as physical displacement or movement. We term “stimulus-responsive colloids” as those that respond by exhibiting changes in their physical characteristics, including swelling, shrinking, or shape-shifting. These terms are not mutually exclusive; a colloid can possess qualities of both active and stimulus-responsive types. We follow this discussion of active and stimulus-responsive colloids by highlighting dynamic colloids created by the Weck group (Figure 3).

### Active Colloids

This section details various active particles, their composition and dynamic behavior including photocatalysis and self-difffusiophoresis^9,5,10,35^ which use chemical reactions to generate propulsion.

Introducing a photocatalytic component into a particle is a common method of altering the properties of colloidal particles. When photocatalytic colloidal particles are exposed to light, electron–hole pairs are created, triggering the decomposition of the photocatalyst surface resulting in particle propulsion.^9^ When exposed to light with energy equal to or greater than its bandgap energy, electrons are excited from the valence band to the conduction band creating electron–hole pairs that are highly reactive and participate in various chemical reactions. For example, a peanut-shaped hematite particle embedded in a 3-(trimethoxysilyl) propyl methacrylate (TPM) sphere, a colloidal “surfer” (Figure 1A), demonstrates propulsion when in a hydrogen peroxide solution.^9^ When optically stimulated by a light source with an energy higher than the bandgap of the hematite, it triggers the decomposition of the surrounding hydrogen peroxide to produce osmotic self-propulsion. Additionally, anisotropic heterodimers of TPM-hematite (Figures 1B–D) demonstrate the same type of photocatalytic self-propulsion.^7,15^ Using blue light with energy higher than the bandgap of the hematite activates the propulsion, while red light with energy lower than the band gap creates an “optical trap” for the particles. This anisotropy extends to Janus (two distinct faces) particles made of different metallic materials with distinct bandgaps. When optically stimulated, both faces of the particles exhibit distinct behaviors. For example, a Janus particle consisting of gold and titania (Figure 1E) in a hydrogen peroxide solution undergoes photocatalytic decomposition to produce motion. When exposed to UV light, the particle maintains a propulsion in the direction of the titania side and propulsion in the direction of the gold side when stimulated by green light.^5^ Also reported is a “Janus motor” with similar particle morphology of a thin platinum layer acting as caps on spherical silica particles.^36^ Janus particles with rod-like morphologies generate propulsion differently than their spherical counterparts using a range of slow and fast motion.^6^ The depiction of a diverse set of active particles with rod-like morphology is shown in Figure 1F. The phoretic activities are influenced by the difference in particle morphology specifically the platinum to gold ratios of these rod-like Janus particles. The morphologies have recorded more challenges compared to their spherical counterparts due to reported aggregations during phoresies.^37^

Particle motion can also result from electro-hydrodynamic flow induced by an electric field of dielectric particles.^8^ The polarization difference resultant from chemical anisotropy influences the orientation and directionality of the particles in an AC electric field.^12^Figure 1G shows particles made via sequential capillarity-assisted particle assembly fabrication method (sCAPA) whose geometry is determined by the template used. These particles can be made from a variety of materials (poly(styrene), silica, titania, etc.) with movement in an electric field based on shape and chemical anisotropy of the particles.^6^ The concept of shape and chemical anisotropy affecting particle behavior in an electric field also extends to patchy particles, particles with regions or patches on their surface possessing distinct chemical or physical properties. Dipatch particles with reduced symmetries (Figure 1H) and gold coated patches exhibited a dielectric matrix resulting from the gold patches creating both chemical and structural anisotropy.^22,38^ The patch size and aspect ratio influence the orientation and directionality of active colloids. The induced-charge electrophoresis mechanism causes these particles to brake and steer with controllable speed.^11^

Particle–particle interactions guided by the presence of dipole–dipole attraction between ferromagnetic colloidal particles can be induced by magnetic fields. Figure 1I shows particles with a cubic morphology where one face of the particle is coated with cobalt.^14^ A magnetic field is used to align the particles. Two distinct responses are observed when the external magnetic field is removed: the formation of a rigid link caused by the partial overlap of the adjacent metallic patches of the cube, resulting in dipole-field attraction, and the magnetic interaction energy between similar metallic patches due to the residual dipole–dipole interactions. The dipole-field attraction between the ferromagnetic cobalt patches on the cubes dominates and becomes responsible for the reversible self-configuration of the cubic particles, resulting in residual dipoles even when the field is removed. The interaction energy between the residual dipoles causes self-folding of the doublet along the common vertex. Therefore, this self-folding mechanism allows the microcube chains to rearrange themselves into stable structures, thereby achieving the desired self-reconfiguration phenomenon.^14^

Active particles are becoming increasingly versatile, owing to their unique properties and chemical composition that can be used to create various materials. Their ability to exhibit collective behavior and autonomous motion opens new avenues for understanding complex systems and designing novel materials such as colloidal machines. Unlike traditional colloidal suspensions, where particles are passively dispersed in a medium, active colloids dynamically interact with each other and their environment. This complex interplay between particle–particle and particle-environment interactions gives rise to emergent phenomena, such as self-assembly.

### Stimulus-Responsive Colloids

A growing body of literature highlights the importance of colloidal materials that respond to external stimuli.^18,19^ This section describes the various stimulus-responsive colloids that exhibit a change in their characteristics as a result of external stimuli, such as chemical, optical, electric, thermal, or magnetic stimulation. These responses include an intrinsic change in subunits, such as swelling, shape-shifting, or change in polarity.

Colloidal particles can respond to optical stimulus via a photocatalytic reaction, creating a phoretic “pump” as seen in the colloidal microcapsules (Figure 2A) that can capture, concentrate, store, and deliver microscopic payloads. As a result of the photocatalytic reaction, a chemical gradient forms internally and nanoparticles near the microcapsules pore are driven inside the capsule. The nanoparticles within the microcapsule are maintained or expelled by altering the pH of the solution.^17^ There is potential for a plethora of time-dependent autonomous micromachinery to be created with this mechanism due to its tunable micropores for uses such as drug delivery.

Colloidal droplets can be coated with DNA strands driving the formation of colloid droplet chains (Figure 2B).^16^ This is achieved with the use of DNA with sticky ends that will bind only to their direct complement. These bonds are thermally regulatable by modulating the temperature to induce particle assembly or disassembly. The droplets (Figure 2B) are coated with several different DNA strands, each with distinct melting temperatures. Control of the system’s temperature facilitates the colloidal droplet chain in folding due to the DNA strands utilized. As DNA sequences are activated, the interactions reveal various geometric possibilities.^16^

Similarly, DNA strands can be incorporated into colloidal patchy particles to facilitate directional bonding. Figure 2C shows a Janus particle, a colloid with two distinct faces, each face is coated in a different DNA sequence.^40^ Each sequence has a distinct melting temperature so that by modulating the temperature the thermoresponsive DNA patches alternate activated sides, creating a system of selective activation and deactivation.

Metallodielectric colloidal particles with gold-coated patches can be induced into various structures (Figure 2D). The electrical fields reorient patches on the colloidal particle, allowing structures of various geometries to be formed. Assembly geometry can be controlled by varying the field strength and direction.

Capillary forces between the oil phase and solid colloidal substrates can be used to engineer shape-shifting particles. This is often induced by pH change and optical stimuli of the stimulus-responsive polymers used in particle fabrication.^18^ For example, monodisperse hematites (cube-, disc-, and peanut-shaped), silica, and titania colloids were used to demonstrate that capillary forces can be altered to cause dewetting and produce shape-shifting particles. Chemical and optical stimuli can be used as triggers to induce the dewetting process. For the peanut-shaped hematite in Figure 2E, introducing blue or green light in the presence of hydrogen peroxide induces pH-triggered dewetting, resulting in a change in particle shape.^18^Figures 2F and G show the corresponding morphological change caused by introducing optical stimuli. The main mechanism for morphological change is the photo-Fenton reaction in which the hydrogen peroxide reacts with the iron in the hematite in the presence of light, forming radicals.^18^ Although harsher conditions are required for similar results when other seed particles such as titania are employed, both triggering mechanisms provide a promising outlook on the potential for customizing dynamic colloids with specific responsiveness to stimuli.

The Weck group has been working toward designing and customizing colloids with intricate architectures and functional capabilities. We developed colloidal particles that respond to changes in the solvent by swelling and deswelling. The solvent-induced shifting is largely attributed to the chemical composition of the particles, in which poly(styrene) (PS) patches respond differently to the solvent environment than the middle TPM belt.^19^ These PS-TPM patchy particles shift between concave and convex features based on cycling chemical stimuli (Figure 3A and B). Additionally, we synthesized DNA-functionalized dipatch particles with ellipsoidal morphology (Figure 3C). Site-specific functionalization with palindromic DNA allowed for the formation of complex periodic structures mediated by patch–patch interactions. The particle design allows for assembly into intricate 2D-colloidal superstructures for potential expansion in colloidal machines.

This section provides a summary of active and stimulus-responsive dynamic colloids. A systematic understanding of how dynamic colloids contribute to the emergence of colloidal machines, however, is still lacking. Following the development of facile protocols for controlling colloidal attributes, the field is exploring the assembly of dynamic particles as the next step in advancement.

## Assemblies of Dynamic Colloids

The focus of this section is on the assemblies of the dynamic particles. Basic assembly strategies from static to dynamic systems use many of the same pathways. Above, we discussed dynamic subunits and the design principles of selecting which type of dynamic component to introduce based on the type of control suitable for a desired assembly. Here, we discuss how these assemblies can be used as components in machines such as actuators, switches, pulleys, levers, sensors, or gears.

### Stimulus Responsive

There are a variety of stimuli that can be used to induce assembly or a change in assembly: chemical, thermal, optical, magnetic, and electric. Stimulus-responsive assemblies are those in which subunits change shape or their assemblies change configuration when stimulated by an external stimulus.

The self-assembly of DNA-coated isotropic spherical colloids has been extensively studied.^2,41,42^ These assemblies generally lead to various closed-packed crystal structures.^2^ To access open-packed structures or structures not at equilibrium, DNA coating has been applied to anisotropic subunits (Janus particles, patchy particles, and clusters) to assemble into complex superstructures. One can further translate the DNA strategy to dynamic assemblies by incorporating multiple DNA strands with different melting points to order/reorder the assembly with a thermal stimulus. Because of the ability to preprogram geometries, this assembly approach has potential applications in colloidal machines (actuator, switch, pulley, lever, sensor, gears), making them ideal for machines that use thermal stimulus as a control handle or trigger.

**Figure 4:**
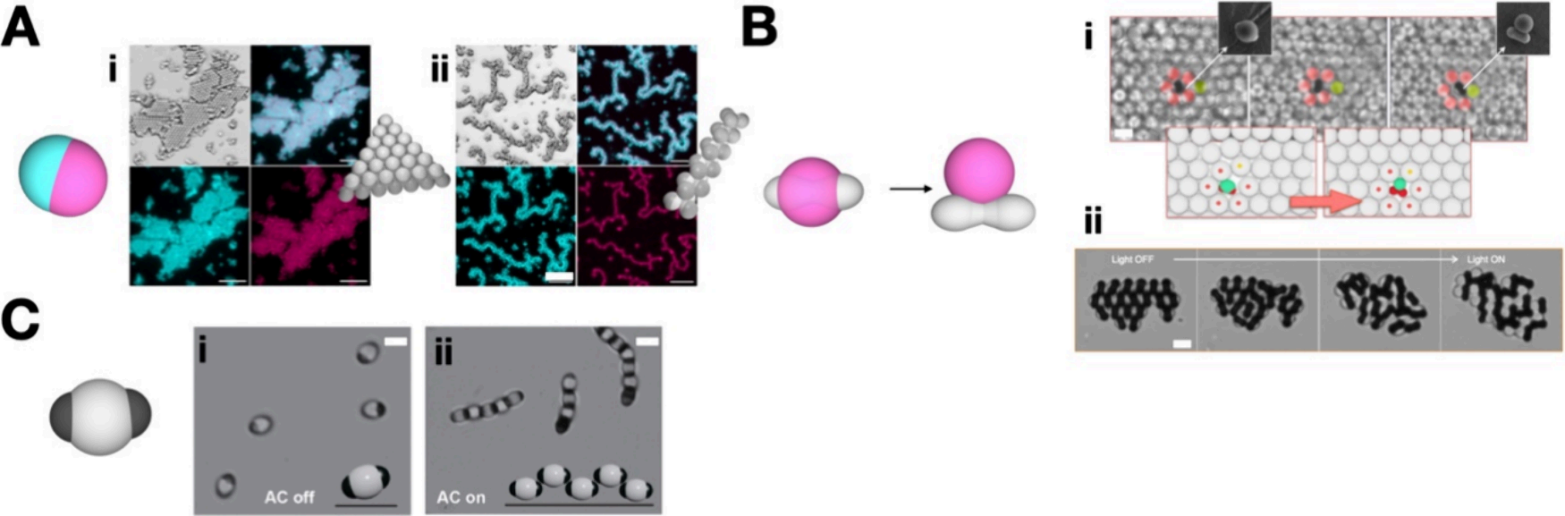
Stimulus responsive assemblies. Assemblies are mediated by DNA interactions and thus responsive to temperature: (A) uses a DNA toehold displacement to activate/deactivate a face of the Janus particles selectively. Reproduced with permission from ref (33). Copyright 2020 American Chemical Society. (B) uses an optical stimulus to cause dewetting and thus a shape change is observed. Reproduced with permission from ref (18). Copyright 2016 The Authors. (C) uses electrical stimulus to induce reconfigurable assemblies such as chains and 2D structures directed by the patches and modulated by tuning the field. Reproduced with permission from ref (12). Copyright 2021 American Chemical Society. Scale bar (A), 10 μm; (B,C), 2 μm.

When using DNA for self-assembly, introducing a toehold or strand displacement allows for the engineering of additional transitions into the assembly without further alterations. This is exhibited in Figure 4A showing the use of DNA Janus particles to fabricate reconfigurable one- and two-dimensional structures via a thermal stimulus. Each face of the Janus particles is coated with a different palindromic DNA strand in combination with toehold strand displacement to activate and deactivate one face independently, thus allowing the assembly to occur between individual faces at a time. The toehold displacement DNA strands are used as a competing interaction. When the temperature is altered, the DNA on one patch is deactivated, and the DNA on the opposite patch is activated. This activation–deactivation in response to a thermal stimulus results in the reconfiguration of the assembly from bilayers to chains. This strategy can be useful for constructing complex systems that reconfigure to assemble into different structures in different environments. With the drastic shape change exhibited, we see them being used as pulley-like machines capable of changing the direction of an internal force or other colloidal component within a colloidal machine. While thermal stimulus is useful for some systems, its requirement for precise thermal control of the assembly makes them nonideal for biomedical applications. In environments where precise thermal control can be achieved, however, one can program in many transitions based on the DNA’s melting point.

In systems where the introduction of heat is not viable or cannot be controlled precisely, other stimuli are necessary. Figure 4B shows the assembly of shape-shifting colloids using dewetting forces between an oil phase (pink) and the solid colloidal substrate (gray) upon introduction of optical stimulus. Figure 4Bi (left) shows the incorporation of the shape-shifting particle into a hexagonal lattice and the subsequent strain imposed on the lattice (bottom left), as seen by the deformation in the lattice packing. Figure 4Bi (right) shows that after stimulation with light, the particles shape-shift, and the lattice strain is relieved (bottom right). Shape-shifting particles such as these can be used within colloidal machines as a lever or switch to alter the shape with stimulus. Figure 4Bii represents an assembly of solely shape-shifting particles and the subsequent change in the morphology of the assembly when an optical stimulus is applied. Again, this shape change and subsequent assembly expansion after irradiation show potential for using these materials as pulleys, actuators, or switches.

In systems where neither thermal nor optical stimuli can be employed and in which the subunits are dielectric, an electrical stimulus can be used. These fields have the benefit of being widely tunable. However, fields typically align particles based on overall structural geometry rather than by structural anisotropy. This essentially suppresses any directionality introduced through the structural anisotropy. To circumvent this issue, Figure 4C uses a system of structurally and chemically anisotropic subunits in electrical fields. The field polarizes the metallic gold coated patches, enabling patch–patch interactions perpendicular to the field orientation to form. Modulation of the field induces changes in the assembly configuration from chains (Figure 4Cii) to 2D structures. Reconfigurable dynamic assemblies are achieved by modulation of the electric field and subunit structural and chemical anisotropies working in tandem. As a result, one can access multiple types of colloidal machine. The downside to electric fields is that when the field is removed assemblies degrade back into a colloidal suspension (Figure 4Ci).

### Active

Active assemblies are those in which the subunits interact with an applied force to generate motion. They assemble or form a moving assembly when stimulated by an external force, such as light irradiation or magnetic or electric fields. These active and field-induced assemblies are often active or assembled only when the external field is applied.

**Figure 5:**
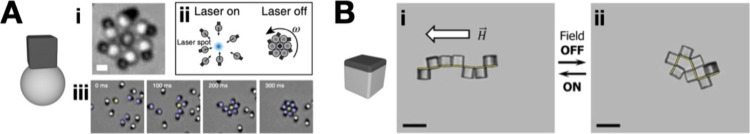
Active and field-induced assemblies demonstrate how various assemblies can form depending on the subunits’ structural/chemical composition and inducing force applied. Here the forces used for the assemblies are (A) optical forming microgears. Reproduced with permission from ref (15). Copyright 2018 The Authors. Scale bar 1 μm. (B) Magnetic microbots. Reproduced with permission from ref (14). Copyright 2017 The Authors. Scale bar 20 μm.

Optical stimulus triggers the targeted formation of self-powered microgears (Figure 5Ai and ii) from active particles forming a rotating superstructure due to diffusiophoresis. The active particles are comprised of hematite that uses hydrogen peroxide as fuel to assemble based on diffusiophoretic interactions. These particles form stable patterns. Upon introducing an optical stimulus, one particle is trapped and pushed down toward the glass, producing a hydrodynamic pumping which further attracts its neighbors, forming the self-propelling assembly (Figure 5Aiii). The angular speed of the assemblies can be tuned with optical intensity. Once the optical stimulus is removed the assemblies remained stable for approximately 20 min. These colloidal microgears have applications in colloidal machines to transmit motion and power within the machine with optical stimulus.

For particles with intrinsic magnetism, magnetic fields can affect dipole interactions to guide the assembly and movement. Figure 5B shows the assembly of metallodielectric monopatch cubes into dynamic chains capable of folding and reconfiguration when a stimulus is applied. When the magnetic field is applied, the magnetic patch on each particle acquires a dipole-inducing long-range attraction. This results in the assembly of chains along the orientation of the applied magnetic field, which can be dynamically rearranged when the stimulus is applied (Figure 5Bii) and removed (Figure 5Biii). The residual polarization by the magnetic field of the metallic facets (dark gray Figure 5Bi) on the patchy cubes leads to interactions between neighboring particles’ patches even once the field is removed. The conformational restrictions of the cubes’ shape and the patch’s location direct this interaction. This uses magnetic field modulation to create microbot clusters with predetermined folding pattern based on the subunits patch orientation (cis/trans) along the chain, creating magnetically controllable and steerable actuators, levers, pulleys, and switches.

The assemblies presented in Figures 4 and 5 represent the current states of colloidal machines. Looking toward the future, we need a clear blueprint of the desired colloidal machine; specifically, focusing on how active and stimulus-responsive colloids will be used and interact with each other within the machine (Table 1). Structure and function of the dynamic colloids in the machine need to be elucidated prior to the machine’s assembly. The key considerations for colloidal machine assemblies begin with solvent systems; all colloidal components need to tolerate the same solvent conditions. Then, we imagine that the integration of multisubunits and stimuli to create functional materials will benefit from a detailed understanding of how these assemblies can work complementarily. Will the subunits work constructively or destructively within the assembly? Are the subunits reactive or inert: to each other, to various triggers required, to products/byproducts resulting from activation by the stimulus? Do the components have distinct and discrete triggers so we can selectively activate a single subunit? Can multiple triggers be integrated into a machine; e.g., what is the difficulty in inserting both an optical and magnetic trigger? How can we make a colloidal machine with structural integrity but with enough flexibility for various applications? Overall, how can we make subunits that are compatible both synthetically and structurally, possessing all the necessary traits to equate to a colloidal machine? Guided by these inquires, one can build the combinatorial design space necessary for the assembly of more complex superstructures.

## Applications

The following section will describe various reported applications for dynamic colloids as described in Table 1. We extrapolate from these reported applications and offer our own interpretation for the potential applications for colloidal machines, ranging from soft robotics and sensors to biomedical applications. For these proof-of-concepts to progress and become real applications, there are many challenges that need to be overcome. One of the most crucial challenges is the need for combinatorial design spaces to be studied in greater detail. By doing so, we can readily access colloidal machines.

### Soft Robotics

**Figure 6:**
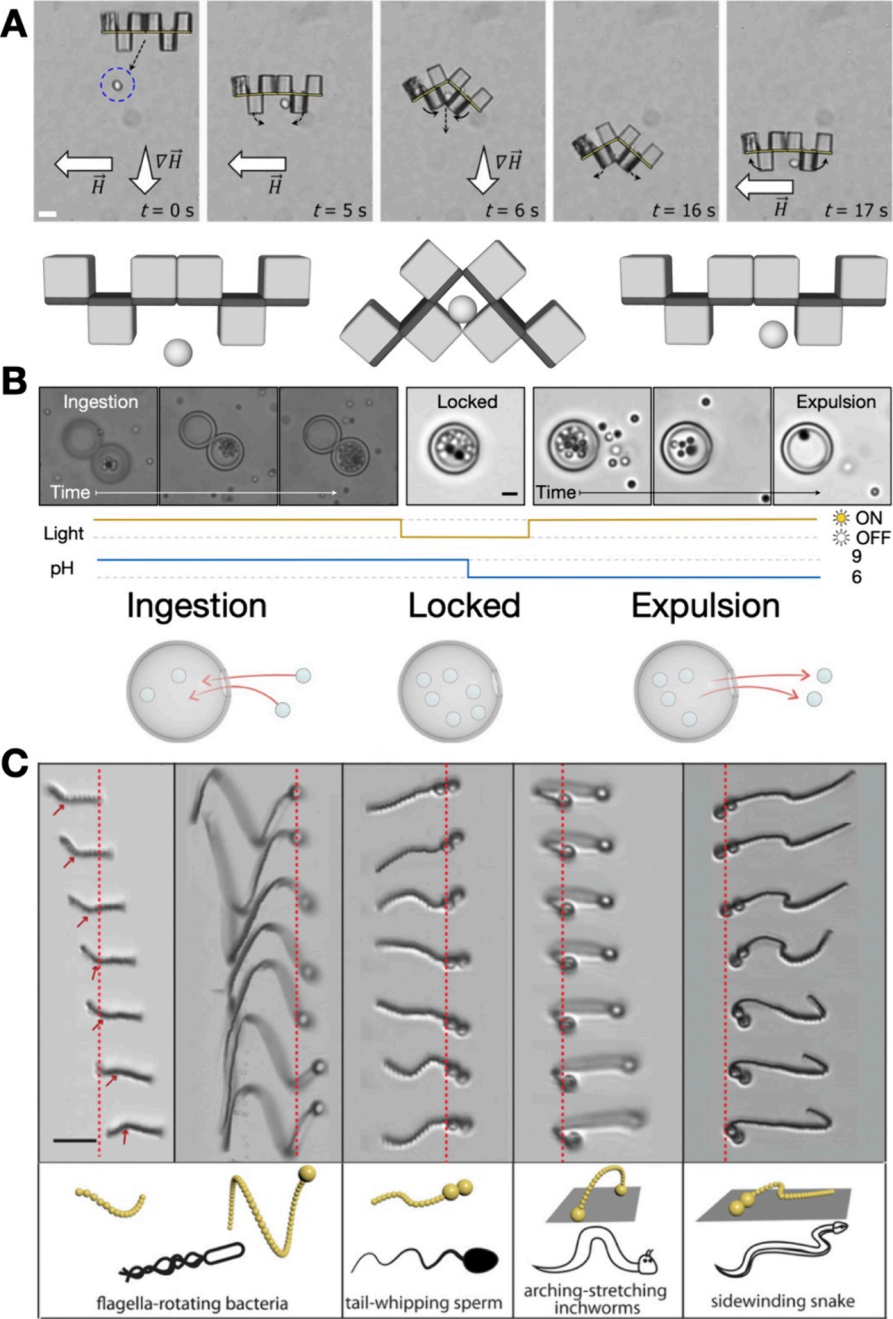
Applications: (A) Soft robot made of Janus cubes capturing a target yeast cell and the subsequent release of the target as the magnetic stimulus is toggled from on to off; Reproduced with permission from ref (14). Copyright 2017 The Authors. (B) Transmembrane transport in inorganic colloidal cell mimics; showing the ingestion of target cargo, locking of cargo, and subsequent expulsion of cargo through optical and chemical stimuli. Reproduced with permission from ref (17). Copyright 2021 Springer Nature. (C) Magnetic chains that mimic natural swimmers and propellers when exposed to undulatory magnetic fields; Reproduced with permission from ref (43). Copyright 2020 The Authors. Scale bar (A) and (C) 10 μm (B) 2 μm.

Soft robots (Figure 6A, C) composed of colloidal subunits have been reported.^44^ These robots have an advantage over conventional robots due to their flexibility and potential for biomimicry. Subunits are ideal for soft robotics due to their dynamic nature and often organic-based building blocks. Their dynamic nature ensures that they can respond to specific stimuli such as light or solvent, while the organic composition can result in inert biocompatible-compliant soft materials. Velev et al. synthesized sequence-encoded colloidal cubes that respond to magnetic stimulus (Figures 5B and 6A).^14^ This particular soft robotic assembly has the potential to capture and transport cargo to a target location.

### Sensors

Colloidal machines can also act as sensors. For example, Janus colloidal motors can be used in analytical sensing of detecting biomolecules^45^ such as cells or bacteria.^46^ When stimulated, the metal face reacts with the solution (catalytically, polarized, etc.), generating a gradient and momentum based on the type of stimulus applied. The second nonmetal face can be functionalized with sensor molecules. After detection, the Janus particles can trap or absorb the target molecule, allowing for detection and targeted removal. Additionally, particles containing metallic components can be used as sensors that can be directed via magnetic fields. This dynamic motion offers an active diffusion method, in contrast to current conventional sensing materials that enable detection or remediation in shorter periods of passive diffusion, making these types of particles potentially more efficient sensors due to their enhanced mobility. In these examples, the colloidal machines have an element of programmability and flexibility that conventional sensors do not have.

### Biomedical Applications

Colloidal machines, when their subunits are synthesized from biocompatible materials, have significant potential in biomedicine. A drug delivery system that mimics cells is shown in Figure 6B. Through a micropore, the system can ingest a target into the internal cavity and expel the target when triggered with optical and chemical stimuli.^17^ In addition to drug delivery, colloidal machines like reconfigurable microbots made from colloidal chains might have other applications in medicine (Figure 6C).^43^ Since these colloidal chains can swim and navigate 3D environments like small capillary blood vessels with a magnetic stimulus, one can imagine colloidal machines with the potential to revolutionize surgical procedures.

## Conclusion

Over the past decade, our ability to synthesize colloidal particles with predesigned shapes, chemical compositions, and morphologies has grown exponentially. Beyond static colloids, the fabrication of dynamic colloids is now a mainstay of the community. Dynamic colloids can be (i) active, i.e., consume fuel, (ii) stimulus-responsive, i.e., change upon the addition of a stimulus, or both. Using a bottom-up design approach allows for specific desired attributes to be incorporated in a controlled manner, resulting in dynamic colloids that can vary in shape, size, and chemical properties, as well as respond to a variety of stimuli. The literature has examples of assemblies of such dynamic colloids into 2D lattices, 2D to 3D shape-shifting assemblies, arbitrary geometries, magnetic colloidal objects, gears, and helical chains.

Colloidal science has seen significant progress in synthesizing dynamic colloids and their assembly; the next frontier is colloidal machines, which are just beginning to advance. The next area of growth will be the formation of multicomponent colloidal machines, where each part reacts differently to different stimuli to cooperatively perform actions. In order to create more complex colloidal machines, one must find ways to combine varied dynamic assemblies to form a singular functional superstructure. By combining multiple types of dynamic colloids with complementary properties, these structures can contain multiple moving and working parts operating in tandem, opening up a whole new world of colloidal applications in various fields, such as biology, environmental engineering, and imaging. By integrating multiple dynamic colloids into one system with distinct methods for stimulation, we can achieve colloidal machines capable of replicating processes carried out on the micro- and nanoscale, bridging the gap between micromachines and molecular machines and allowing researchers to work in an entirely new scale.
